# Changes in Buprenorphine Prescribing to Medicaid Beneficiaries During the First Year of the COVID-19 Pandemic

**DOI:** 10.1001/jamanetworkopen.2022.4058

**Published:** 2022-03-24

**Authors:** William N. Dowd, Tami L. Mark

**Affiliations:** 1 Community Health Research Division, RTI International, Research Triangle Park, North Carolina

## Abstract

This cross-sectional study compares trends in prescribing buprenorphine to all Medicaid beneficiaries before and after the start of the COVID-19 pandemic.

## Introduction

The COVID-19 pandemic posed challenges for access to opioid use disorder treatment, such as buprenorphine. In response, state and federal policy makers reduced in-person interactions required for buprenorphine prescribing,^1,2^ and clinicians issued longer-duration buprenorphine prescriptions.^3^ Whether the combined effect of policy and clinical adaptation was sufficient to offset challenges to buprenorphine access is not well known. To our knowledge, this study is the first to investigate changes in buprenorphine prescribing to all Medicaid beneficiaries during the first year of the pandemic.

## Methods

This cross-sectional study was conducted using publicly available, deidentified data and, as such, did not require institutional review board approval or informed patient consent, in accordance with 45 CFR §46. We used quarterly Medicaid State Drug Utilization Data (SDUD) from 2018 to 2020 to construct 3 measures of buprenorphine provision. The primary measure—units (defined as tablets or sublingual films) dispensed per 1000 beneficiaries (units)—represents total buprenorphine supplied to Medicaid beneficiaries. Two other measures—prescriptions per 1000 beneficiaries (prescriptions) and mean units dispensed per prescription (units per prescription)—decompose total supply into component parts. For each measure, we used linear regression to compute deviation from prepandemic trends during the pandemic—defined as the second through fourth quarters of 2020. For all Medicaid programs combined and for each state separately, we computed cumulative deviations for units and prescriptions, and mean deviations for units per prescription over the pandemic period (eAppendix in the Supplement).

Statistical testing was 2-sided, and *P* < .05 was considered statistically significant. Statistical analysis was conducted using Stata software version 16.1 (StataCorp) from November 2021 to February 2022.

## Results

**Figure 1.**
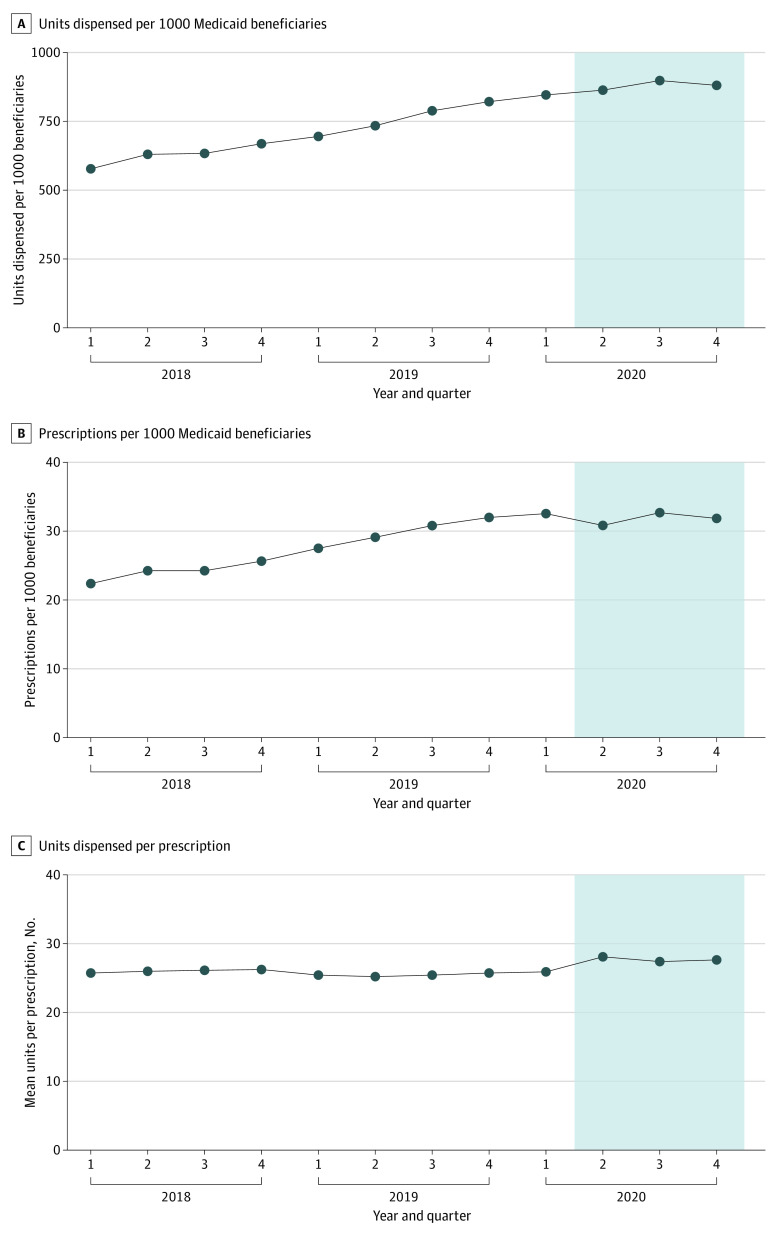
Buprenorphine Prescription Measures for All Medicaid Programs, 2018-2020 Shaded region indicates pandemic period.

Across Medicaid programs prior to the pandemic, units and prescriptions were increasing, while units per prescription was flat. After the start of the pandemic, the trend in units flattened, the trend in prescriptions flattened following an initial drop, and units per prescription increased and then remained elevated (Figure 1). Regression analysis indicated that cumulative units dispensed during the first 3 quarters of the pandemic was below the prepandemic trend by 92.75 per 1000 Medicaid beneficiaries (95% CI, −174.82 to −10.68 per 1000 Medicaid beneficiaries). Cumulative prescriptions issued was also below the prepandemic trend by 11.56 per 1000 beneficiaries (95% CI, −15.42 to −7.71 per 1000 Medicaid beneficiaries), but mean units dispensed per prescription increased by 2.20 per 1000 Medicaid beneficiaries (95% CI, 1.34 to 3.06 per 1000 Medicaid beneficiaries) relative to the prepandemic trend.

**Figure 2.**
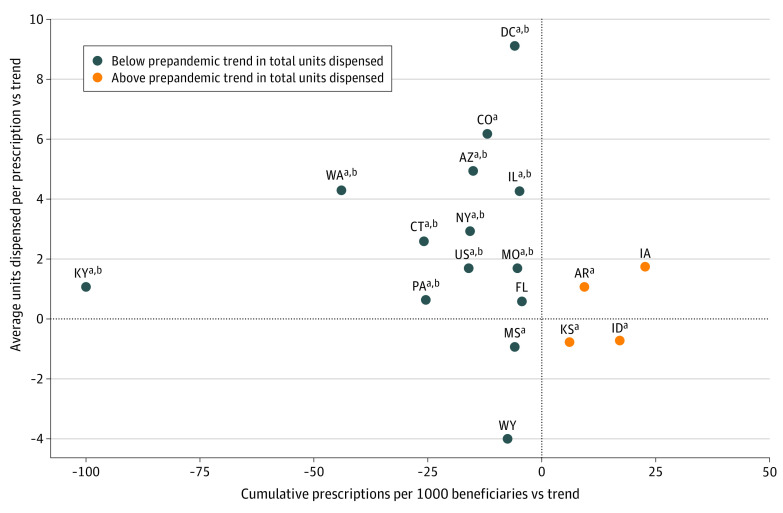
National and State-Level Differences From Prepandemic Trends on 3 Buprenorphine Prescription Measures States without significant differences from trend in units dispensed are not shown. The point labeled US represents the national estimate. AR indicates Arkansas; AZ, Arizona; CO, Colorado; CT, Connecticut; DC, Washington DC; FL, Florida; IA, Iowa; ID, Idaho; IL, Illinois; KS, Kansas; KY, Kentucky; MO, Missouri; MS, Mississippi; NY, New York; PA, Pennsylvania; WA, Washington; WY, Wyoming. ^a^Significant differences in cumulative prescriptions per 1000 beneficiaries during the first 3 quarters of the pandemic compared with the prepandemic trend. ^b^Significant differences in mean units dispensed per prescription during the first 3 quarters of the pandemic compared with the prepandemic trend.

In 13 states, total buprenorphine dispensed during the pandemic was significantly below prepandemic trends, and in 4 states it was significantly above trend (Figure 2). Figure 2 plots cumulative prescriptions (x-axis) and mean units per prescription (y-axis) in these 17 states relative to prepandemic trends. Most states were in the quadrant represented by fewer prescriptions and more units dispensed per prescription relative to prepandemic trends.

## Discussion

Cumulative units of buprenorphine dispensed per 1000 Medicaid beneficiaries was significantly below the prepandemic trend nationally and in 13 states in the first 3 quarters of the COVID-19 pandemic. This was despite increases in units per prescription, which partially offset below-trend prescribing in some states. Previous studies found no change in the number of unique Medicaid beneficiaries receiving buprenorphine prescriptions at the pandemic’s onset,^4^ and noted increases in days’ supply per prescription.^5^ The key finding of below-trend buprenorphine dispensing is particularly concerning given the increase in opioid overdose deaths over the same period.^5,6^

This study has some limitations. First, Medicaid programs submit aggregate rather than individual- or claim-level data. Therefore, we could neither distinguish between new prescriptions and refills nor determine if trend deviations varied across subpopulations. Second, we could not examine whether expanding units per prescription had adverse outcomes, such as more diversion. Third, this study describes associations and does not make any causal statements or attribute findings to specific policies or clinical adaptations. For example, we could not isolate state policy changes—such as relaxation of prescription quantity limits^1,2^—from other factors owing to limited information on the nature of these policies and their specific applicability to buprenorphine. Further research should evaluate whether state-level changes affected quantity per prescription, and whether that mitigated access barriers.

## Notes

Supplement. **eAppendix.** Supplemental Methods
