# Supplementary material for: Changes in Buprenorphine Prescribing to Medicaid Beneficiaries During the First Year of the COVID-19 Pandemic
Source: JAMA Netw Open. 2022 Mar 24;5(3):e224058. doi: 10.1001/jamanetworkopen.2022.4058 (PMC8948527; doi:10.1001/jamanetworkopen.2022.4058)
Supplement: Supplement. — eAppendix. Supplemental Methods [file jamanetwopen-e224058-s001.pdf]

Supplemental Online Content

Dowd WN, Mark TL. Changes in buprenorphine prescribing to Medicaid beneficiaries during the first year of the COVID-19 pandemic. *JAMA Netw Open.* 2022;5(3):e224058. doi:10.1001/jamanetworkopen.2022.4058

eAppendix. Supplemental Methods

This supplemental material has been provided by the authors to give readers additional information about their work.

© 2022 Dowd WN et al. *JAMA Network Open.*

eAppendix. Supplemental Methods

Data

The primary source of data for this analysis is State Drug Utilization Data (SDUD) provided by the Centers for Medicare and Medicaid Services (CMS) and available at https://www.medicaid.gov/medicaid/prescription-drugs/state-drug-utilization-data/index.html. This dataset captures the number of prescriptions issued and units dispensed for all prescription drugs paid for by Medicaid programs. All states are required to submit this information to CMS on a quarterly basis. CMS cautions that data from the most recently reported quarter (quarter 1 of 2021 at the time the analysis was completed) are provisional, so data from the first quarter of 2021 were omitted from this analysis.

All SDUD records representing buprenorphine were identified, and formulations used for treatment of chronic pain were excluded using information from the National Drug Code directory, available at https://www.fda.gov/drugs/drug-approvals-and-databases/national-drug-code-directory. Three measures were constructed: units dispensed per 1000 beneficiaries, prescriptions per 1000 beneficiaries, and average units dispensed per prescription. NDCs representing buprenorphine injections (e.g., Sublocade) were excluded as they could not be compared with oral/sublingual buprenorphine in terms of units dispensed and made up less than 0.5% of total prescriptions over the study period.

Analysis

Ordinary least squares models of the form shown below (equation 1) were estimated for each measure (y_t).

$$y = \beta_0 + \sum_{i=1}^{3}(\beta_i * P_i) + \beta_4 T + \varepsilon \qquad \text{(equation 1),}$$

where P_i represents an indicator for the first, second, and third quarters of the pandemic (ie, quarter 2 through quarter 4 of 2020), T represents continuous time, and ε_t is an error term. The coefficients β_1, β_2, and β_3 are deviations from the pre-pandemic trend. For the units dispensed per 1000 beneficiaries and prescriptions per 1000 beneficiaries measures, the coefficients β_1-$\beta_3$were added up to represent cumulative deviations from trend. For the average units dispensed per prescription measure, the three coefficients were averaged. Confidence intervals around sums and averages were computed using “lincom” in Stata 17. This approach was used for national-level measures (the sum of each measure across all Medicaid programs) and state-level measures.

© 2022 Dowd WN et al. *JAMA Network Open.*
